# VITAMIN D DEFICIENCY AND PARATOHOMMONIUM INCREASE IN LATE POSTOPERATIVE GASTRIC BYPASS IN ROUX-EN-Y

**DOI:** 10.1590/0102-672020180001e1407

**Published:** 2018-12-06

**Authors:** Daniela Vicinansa MÔNACO-FERREIRA, Vânia Aparecida LEANDRO-MERHI, Nilton César ARANHA, Andre BRANDALISE, Nelson Ary BRANDALISE

**Affiliations:** 1 Graduate Program in Health Sciences, Pontifical Catholic University of Campinas; 2 Lane Clinic, Campinas, SP, Brazil

## Abstract

*****Background*** ****:**

Roux-en-Y gastric bypass patients can experience changes in calcium metabolism and hyperparathyroidism secondary to vitamin D deficiency.

*****Aim*** ****:**

To evaluate nutritional deficiencies related to the calcium metabolism of patients undergoing gastric bypass with a 10-year follow-up.

*****Method*** ****:**

This is a longitudinal retrospective study of patients submitted to Roux-en-Y gastric bypass at a multidisciplinary clinic located in the Brazilian southeast region. The study investigated the results of the following biochemical tests: serum calcium, ionized calcium, vitamin D, and parathormone (PTH). The generalized estimating equations (GEE) determined the nutritional deficiencies using a significance level of 5%.

*****Results*** ****:**

Among the patients who finished the study (120 months), 82.86% (n=29) had vitamin D deficiency, and 41.94% (n=13) had high PTH. Postoperative time had a significant effect on PTH (p=0.0059). The percentages of patients with vitamin D, serum calcium, and ionized calcium deficiencies did not change significantly over time.

*****Conclusion*** ****:**

One of the outcomes was vitamin D deficiency associated with secondary hyperparathyroidism. These findings reaffirm the importance of monitoring the bone metabolism of patients submitted to Roux-en-Y gastric bypass.

**HEADINGS:**

Calcium deficiency. Vitamin D deficiency. Secondary hyperparathyroidism.

## Resumo

**Racional:**

Pacientes submetidos ao bypass gástrico em Y-de-Roux, podem apresentar alterações do metabolismo do cálcio e hiperparatireoidismo secundário à deficiência de vitamina D.

**Objetivo:**

Avaliar as deficiências nutricionais relacionadas ao metabolismo do cálcio de pacientes submetidos à bypass gástrico em Y-de-Roux, com seguimento de 10 anos.

**Método:**

Um estudo retrospectivo longitudinal foi conduzido com pacientes submetidos à bypass gástrico em Y-de-Roux, em uma Clínica Multidisciplinar no Sudeste do Brasil. Investigou-se a frequência do acompanhamento médico e nutricional e os exames bioquímicos de cálcio sérico, cálcio iônico, vitamina D e paratormônio (PTH). Para a análise das deficiências nutricionais, foram utilizadas as Equações de Estimativas Generalizadas (EEG), com nível de significância de 5%.

**Resultados:**

Dos pacientes que permaneceram no estudo até o final (120 meses), 82,86% (29), apresentaram níveis de deficiência de vitamina D e 41,94% (13) apresentaram PTH elevado. O efeito do tempo foi significativo para o PTH (p=0,0059). Para a vitamina D, cálcio sérico e cálcio iônico, o percentual de deficiência manteve-se constante ao longo do tempo, sem diferença significativa entre os tempos.

**Conclusão:**

A deficiência de vitamina D, associada ao hiperparatireoidismo secundário, foi um desfecho encontrado. Tais achados reafirmam a importância do cuidado com o metabolismo ósseo, em pacientes submetidos à bypass gástrico em Y-de-Roux.

## INTRODUCTION

Roux-en-Y gastric bypass is the most common gastric bypass surgery in the world ^2^, still ahead of vertical gastrectomy^2^. Bariatric surgery is a safe and effective procedure to treat patients diagnosed with morbid obesity^16^. The results are related to sustained weight loss and the improvement or resolution of comorbidities associated with morbid obesity^5^ ^,^ ^29^. However, nutritional deficiencies stemming from food restriction associated with poor nutrient absorption are important issues to consider in the follow-up of these patients^6^ ^,^ ^16^ ^,^ ^19^ ^,^ ^25^.

The post-operative follow-up is part of the recommendations in bariatric surgery and should be conducted by professionals who live the reality of the bariatric patient^16^; however, the follow-up loss is considered high by studies with long-term follow-ups^5^ ^,^ ^19^. In recent systematic review and meta-analysis, Buchwald et al^5^, evaluated the results after gastric bypass and described limited follow-up rates as conclusion.

Roux-en-Y gastric bypass patients can experience changes in calcium metabolism and hyperparathyroidism secondary to vitamin D deficiency ^1^ ^,^ ^10^ ^,^ ^16^, requiring that professionals who follow these patients pay close attention to these items and use effective instruction and monitoring strategies^16^.

Many studies^1^ ^,^ ^7^ ^,^ ^10^ ^,^ ^28^ ^,^ ^33^ ^,^ ^34^ have assessed bone loss after Roux-en-Y gastric bypass and described changes in bone mineral density caused by many factors related to hyperparathyroidism secondary to vitamin D deficiency and dramatic weight loss. More recently, studies have found hormonal and metabolic changes in this population, which can affect bone homeostasis^4^.

In a 24-month prospective study, Muschitz et al, 2016 ^18^ found that vitamin D, calcium, and protein supplementation associated with physical exercises slow the loss of bone mineral density after bariatric surgery^18^.

Bariatric surgery guidelines^16^ advise all patients to take calcium and vitamin D supplements and to undergo biochemical tests regularly to monitor their metabolic profile.

Considering the importance of nutritional monitoring and the treatment of nutritional deficiencies related to the surgical procedure, the objective of this study was to evaluate nutritional deficiencies related to the calcium metabolism of patients undergoing gastric bypass with a 10-year follow-up.

## METHOD

All procedures performed in studies involving human participants were in accordance with the ethical standards of the institutional and/or national research committee and with the 1964 Helsinki declaration and its later amendments or comparable ethical standards. This study is part of a larger project (master’s research project) approved by the institution’s Research Ethics Committee. 

This is a retrospective longitudinal study collected medical and nutritional data from the medical records of Roux-en-Y gastric bypass patients ten years after the surgery. These patients visited a multidisciplinary clinic located in the Brazilian southeast region between January 2005 and May 2015. The study inclusion criteria were having undergone laparoscopic unbanded Roux-en-Y gastric bypass and having attended the medical and nutritional follow-ups in the first 12 months after surgery. The exclusion criteria were patients submitted to other surgical techniques or who had not attended the medical and nutritional follow-ups regularly in the first year after surgery. Thus, the study included 106 patients. 

### Data collection 

The results of biochemical tests, namely serum calcium, ionized calcium, parathormone (PTH), and vitamin D, were collected from the patients’ medical and nutritional records. The study occasions were immediately before surgery and 3, 6, 12, 24, 48, 72, 96, and 120 months after surgery. 

### Biochemical tests 

The biochemical tests included serum calcium, ionized calcium, vitamin D, and parathormone (PTH). All results were recorded preoperatively and 3, 6, 12, 24, 48, 72, 96, and 120 months after surgery. Calcium and vitamin D deficiencies were classified as recommended by the Institute of Medicine, 2011^13^. Serum calcium was considered deficient when below 8.5 mg/dl^13^. Vitamin D was considered deficient when below 20 ng/ml, and insufficient when between 21 and 29 ng/ml. Ionized calcium^9^ was considered deficient when below 1.12 mmol/L. PTH was considered high when above 65 pg/ml^32^, which was also the criterion used for classifying secondary hyperparathyroidism^32^. 

### Nutritional counseling 

All patients were instructed on the importance of clinical and nutritional follow-up after the surgical procedure. The patients received dietary guidance and were instructed to take multivitamin supplement, iron chelate, injectable vitamin B12, calcium chelate and vitamin D, with individually adjusted doses, according to routine exams to evaluate the metabolic profile during nutritional follow-up.

### Statistical analysis 

Data were tabulated in the software Excel®, and the statistical analyses were performed by the software SPSS v.10.0. The nominal variables were expressed as percentages. The generalized estimating equations (GEE) compared proportions over time using a significance level of 5%^23^ ^,^ ^24^. In vitamin D analysis, insufficiency and deficiency were grouped as deficiency. 

## RESULTS

Table 1 shows the results of the calcium serum, ionized calcium, PTH, and vitamin D tests. Only the patients who underwent the biochemical tests in each study occasion were included in the analysis of that occasion. Preoperatively, 103 patients (97.17%) had normal serum calcium level; nine patients (14.29%) had ionized calcium deficiency; and seven (9.46%) high PTH (Table 1).

**TABLE 1:** Prevalence of nutritional deficiencies associated with calcium metabolism in patients submitted to Roux-en-Y gastric bypass over a 10-year period

Biochemical tests	Time								
	Pre-op n (%)	3 months n (%)	6 months n (%)	12 months n (%)	24 months n (%)	48 months n (%)	72 months n (%)	96 months n (%)	120 months n (%)
Serum calcium * (p=0.1083)									
Sufficient	103 (97.17)	89 (90.82)	60 (89.55)	101 (93.52)	92 (92.93)	78 (87.64)	61 (93.85)	38 (88.37)	36 (97.3)
Deficient	3 (2.83)	9 (9.18)	7 (10.45)	7 (6.48)	7 (7.07)	11 (12.36)	4 (6.15)	5 (11.63)	1 (2.7)
Ionized calcium * (p=0.5995)									
Sufficient	54 (85.71)	68 (86.08)	53 (92.98)	86 (89.58)	73 (86.90)	69 (84.15)	53 (85.48)	37 (90.24)	28 (87.5)
Deficient	9 (14.29)	11 (13.92)	4 (7.02)	10 (10.42)	11 (13.10)	13 (15.85)	9 (14.52)	4 (9.76)	4 (12.5)
Parathormone * (p=0.0059)									
Normal	67 (90.54)	66 (86.84)	43 (81.13)	76 (79.79)	69 (77.53)	67 (72.83)	44 (67.69)	27 (65.85)	18 (58.06)
High	7 (9.46)	10 (13.16)	10 (18.87)	19 (20.21)	20 (22.47)	26 (27.17)	21 (32.31)	14 (34.15)	13 (41.94)
Vitamin D * (p=0.0829)									
Sufficient	**	18 (48.65)	10 (47.62)	18 (42.86)	21 (38.89)	14 (31.82)	17 (41.46)	8 (23.53)	6 (17.14)
Deficient	**	19 (51.35)	11 (52.38)	24 (57.14)	33 (61.11)	30 (68.18)	24 (58.54)	26 (76.47)	29 (82.86)

Time=follow-up time in months; Pre-op=preoperative; patients with vitamin D insufficiency and deficiency were grouped together and considered deficient;

*Generalized estimating equations (GEE) using a significance level of 5%;

** pre-operative assessment not included because most patients had incomplete data, preventing analysis.

Six months after surgery, 7 (10.45%) patients had serum calcium deficiency; 4 (7.02%) had ionized calcium deficiency; 10 (18.87%) had high PTH; and 11 (52.38%) had vitamin D deficiency (Table 1). 

In the 24-month follow-up, 7 (7.07%) patients had serum calcium deficiency; 11 (13.10%) ionized calcium deficiency; 20 (22.47%) high PTH; and 33 (61.11%) vitamin D insufficiency or deficiency (Table 1).

In the 72-, 96-, and 120-month follow-ups, 4 (6.15%), 5 (11.63%), and 1 (2.7%) patients, respectively, had serum calcium deficiency; 9 (14.52%), 4 (9.76%), and 4 (12.5%), respectively, had ionized calcium deficiency; 21 (32.31%), 14 (34.15%), and 13 (41.94%), respectively, had high PTH; and 24 (58.54%), 26 (76.47%), and 29 (82.86%) had vitamin D deficiency (Table 1). 

Postoperative time affected PTH significantly (p=0.0059). The percentages of patients with vitamin D, serum calcium, and ionized calcium deficiencies remained constant over time (Table 1).

**FIGURE 1:**
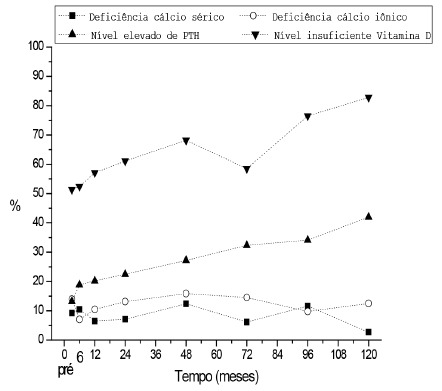
Prevalence of nutritional deficiencies associated with calcium metabolism over time, according to the generalized estimating equations (GEE)

Figure 1 shows the prevalence of nutritional deficiencies, according to the generalized estimating equations, associated with calcium metabolism over time.The results demonstrated a constant deficiency of vitamin D and increase of the PTH, with the maintenance of the serum levels of calcium ionic and serum calcium within the limits of the normality. These results demonstrated that the prescribed supplements were not enough for the treatment of the deficiencies.

## DISCUSSION

One of the great challenges of patients under bariatric surgery refers to the long-term follow-up in the postoperative period. Although patients are advised to perform regular follow-up with the multidisciplinary team, follow-up loss is significant in obesity treatment centers. The results found in this study regarding medical and nutritional monitoring also demonstrate this reality, being an important complicating factor for the diagnosis and treatment of nutritional deficiencies.

Regardless of the metabolic benefits stemming from gastric bypass^5^, many studies ^11,14,19,26,27,31^ have reported the impact of nutritional deficiencies after gastric bypass, including its effects on bone metabolism ^1^ ^,^ ^4^ ^,^ ^7^ ^,^ ^8^ ^,^ ^10^ ^,^ ^17^ ^,^ ^18^ ^,^ ^22^ ^,^ ^28^ ^,^ ^30^ ^,^ ^33^ ^,^ ^34^.

Because of the importance of bone metabolism and the nutritional and metabolic implications of gastric bypass on health, the present study investigated changes in the biochemical parameters of calcium metabolism. 

One of the great challenges related to gastric bypass surgery is long-term patient monitoring after surgery. The results of many studies^1,11,14^ confirm this reality. 

The challenge of long-term monitoring may compromise clinical and nutritional management, and nutritional deficiencies often develop and/or become more severe. The treatment protocol of these chronic patients should include making them aware of the importance of attending follow-up visits and the metabolic consequences of nutritional deficiencies. 

Regarding nutritional deficiencies, the results of many studies ^1,4,7,8,10,14,17,18,22,28,30,33,34^ have indicated the importance of monitoring bone metabolism after gastric bypass by measuring biochemical parameters and providing adequate calcium, vitamin D, and protein supplementation ^16^ ^,^ ^18^ ^,^ ^30^.

One of the clinical outcomes of the present sample was hyperparathyroidism secondary to vitamin D deficiency in the late postoperative period ^**3**^. This study, which included men and women, found that roughly 42% of the patients had high PTH in the 10-year follow-up. Yet, time had no effect on serum calcium, ionized calcium, and vitamin D. Serum calcium and ionized calcium did not change over time. 

El-Kadre *et al* ^10^ published similar findings in 2004: high PTH and normal serum calcium. They studied calcium metabolism in morbidly obese pre- and postmenopausal women submitted to Roux-en-Y gastric bypass and found changes in the calcium metabolism of both groups. Serum calcium did not change in either group. The authors suggested that all patients submitted to Roux-en-Y gastric bypass should take calcium and vitamin D supplements^10^. More recently, the Bariatric Surgery Guidelines^*2*^ also suggested that all patients submitted to Roux-en-Y gastric bypass should take calcium and vitamin D supplements ^10,16^.

The present study measured PTH on different occasions over the 10-year postoperative period and found that PTH changed significantly over time. Many studies^1^ ^,^ ^10^ ^,^ ^14^ ^,^ ^22^ that investigated the effect of time on PTH made similar findings. In a longitudinal study about the effect of gastric bypass on bone density, vitamin D, and PTH five years after surgery, Raoof *et al*.^22^ found a significant increase of PTH over time, also corroborating the present study.

Assessment of the prevalence of nutritional deficiencies in the present study in various follow-up visits over a ten-year period showed that PTH varied significantly. PTH had increased significantly 12, 24, 48, 72, 96, and 120 months after surgery. Karefylakis *et al* ^14^ assessed vitamin D status and PTH 10 years after gastric bypass and found secondary hyperparathyroidism as the clinical outcome, with 65% and 69% of the patients presenting vitamin D deficiency and high PTH, respectively. 

A recent study^17^ on nutritional status, body composition, and bone health in women after gastric bypass found high PTH and low vitamin D in women with higher postoperative time, similar to the present findings.

In Brazil Costa *et al.,* 2016^8^ found secondary hyperparathyroidism in 41.7%, vitamin D insufficiency and deficiency in 83.8%, and hypocalcemia in 14.3% of their sample, but normal levels of magnesium and phosphorus. The present study found very similar results in the 10-year follow-up: 41.94% of the sample had high PTH, 82.86% had vitamin D deficiency, 12.5% had hypocalcemia, and most patients had normal magnesium and phosphorus levels. 

Vitamin D is an essential nutrient that acts on the homeostasis of bone metabolism^12^. A high prevalence of vitamin D insufficiency was observed in bariatric patients, but prospective studies are still needed^15^. Low vitamin D may be related to high vitamin D storage. The fat-soluble nature of vitamin D may allow its immobilization by adipose tissue, and initial weight loss would release vitamin D from the adipose tissue^15^. 

A limitation of the present study is its retrospective design, which prevents assessment of vitamin D deficiency and other determinants in a control group, such as dietary intake, calcium and vitamin D supplementation, and sun exposure. 

In a prospective cohort study, Lin *et al.,* 2011 ^15^, measured plasma vitamin D 24 months after gastric bypass as they were concerned with vitamin D and the implications on bone metabolism. They found a high prevalence of vitamin D deficiency and confirmed that gastric bypass worsened vitamin D status. 

The present study assessed both vitamin D insufficiency and deficiency over time. Vitamin D deficiency was found in roughly 83% of the patients, but this prevalence did not change significantly over time. Many authors^8^ ^,^ ^14^ ^,^ ^15^ reported similar results, with vitamin D deficiency present in 60 to 80% of patients submitted to Roux-en-Y gastric bypass. 

The mechanisms involved in bone loss after gastric bypass regard hyperparathyroidism associated with vitamin D deficiency and rapid weight loss. Recently, other possibilities have also been investigated, such as the influence of hormonal changes on subjacent mechanisms that may contribute to bone loss^20^ ^,^ ^21^. More studies are necessary to understand all variables that affect calcium metabolism after gastric bypass. However, long-term results show the importance of paying attention to these mechanisms in the follow-up of these patients. 

A positive aspect of the present study was the possibility of assessing biochemical parameters over a postoperative period of 10 years. Another limitation was the impossibility of assessing primary hyperparathyroidism, which would have been used as an exclusion criterion. 

Different results from the present study were found by Muschitz *et al*., 2016^18^, who assessed the impact of vitamin D, calcium, protein supplementation, and physical exercise two years after gastric bypass. The authors found that vitamin D and protein supplementation associated with physical exercise decreased the loss of bone mineral density^18^. 

Worn _^*et al., 2015 30,*^_ investigated blood changes related to calcium metabolism two years after Roux-en-Y gastric bypass, but their results were very different from those in recent literature; they attribute their positive results to regular postoperative follow-up visits and individual supplementation adjustments. Those authors found that vitamin D levels increased in males and females, and they did not find secondary hyperparathyroidism in the two-year follow-up. Their results showed that calcium and vitamin D supplementation was all that their sample needed to prevent loss of bone mineral density. 

The high prevalence of vitamin D deficiency, associated with elevated PTH over a 10-year follow-up, suggests that the dosages of calcium and vitamin D supplements prescribed were not enough for prevention and treatment in the studied population. A careful evaluation of the calcium metabolism in patients under long-term gastric bypass is necessary, considering the nutritional prescription and the intake of the supplements in dosages capable of reversing and controlling the nutritional deficiencies of the calcium metabolism.

These results confirm the importance of medical and nutritional follow-up after Roux-en-Y gastric bypass and of having a multidisciplinary team capable of monitoring these patients and providing appropriate vitamin and mineral supplements. 

The limitations of this study include the number of patients lost to the 10-year follow-up and study design: its retrospective nature prevented the assessment of other variables considered important for the study outcomes. On the other hand, this study provides an important contribution because of the long-term assessment and the scarcity of longitudinal studies with long-term follow-ups. We can also consider as limitations the impossibility to evaluate the control patients for vitamin D deficiency.

## CONCLUSION

One of the outcomes was vitamin D deficiency associated with secondary hyperparathyroidism. The nutritional counseling was not enough to reverse cases of nutritional deficiencies. These findings reaffirm the importance of monitoring the calcium metabolism of patients submitted to Roux-en-Y gastric bypass. 
